# Quantitative thresholds for variant enrichment in 13,845 cases: improving pathogenicity classification in genetic hearing loss

**DOI:** 10.1186/s13073-023-01271-7

**Published:** 2023-12-18

**Authors:** Sihan Liu, Mingjun Zhong, Yu Huang, Qian Zhang, Ting Chen, Xiaofei Xu, Wan Peng, Xiaolu Wang, Xiaoshu Feng, Lu Kang, Yu Lu, Jing Cheng, Fengxiao Bu, Huijun Yuan

**Affiliations:** 1 Department of Oto-Rhino-Laryngology, West China Hospital, Sichuan University, Chengdu, 610000 China; 2 Institute of Rare Diseases, West China Hospital, Sichuan University, Chengdu, 610000 China

**Keywords:** PS4, ACMG/AMP guidelines, Variant classification, Cohort dataset, Hearing loss

## Abstract

**Background:**

The American College of Medical Genetics and Genomics (ACMG)/Association for Molecular Pathology (AMP) guidelines recommend using variant enrichment among cases as "strong" evidence for pathogenicity per the PS4 criterion. However, quantitative support for PS4 thresholds from real-world Mendelian case–control cohorts is lacking.

**Methods:**

To address this gap, we evaluated and established PS4 thresholds using data from the Chinese Deafness Genetics Consortium. A total of 9,050 variants from 13,845 patients with hearing loss (HL) and 6,570 ancestry-matched controls were analyzed. Positive likelihood ratio and local positive likelihood ratio values were calculated to determine the thresholds corresponding to each strength of evidence across three variant subsets.

**Results:**

In subset 1, consisting of variants present in both cases and controls with an allele frequency (AF) in cases ≥ 0.0005, an odds ratio (OR) ≥ 6 achieved strong evidence, while OR ≥ 3 represented moderate evidence. For subset 2, which encompassed variants present in both cases and controls with a case AF < 0.0005, and subset 3, comprising variants found only in cases and absent from controls, we defined the PS4_Supporting threshold (OR > 2.27 or allele count ≥ 3) and the PS4_Moderate threshold (allele count ≥ 6), respectively. Reanalysis applying the adjusted PS4 criteria changed the classification of 15 variants and enabled diagnosis of an additional four patients.

**Conclusions:**

Our study quantified evidence strength thresholds for variant enrichment in genetic HL cases, highlighting the importance of defining disease/gene-specific thresholds to improve the precision and accuracy of clinical genetic testing.

**Supplementary Information:**

The online version contains supplementary material available at 10.1186/s13073-023-01271-7.

## Background

The advancement of high-throughput sequencing technologies has greatly enhanced genetic testing capabilities and holds immense potential for personalized medical management. To bring clarity in the clinical interpretation of genetic variants, the American College of Medical Genetics and Genomics (ACMG) and the Association for Molecular Pathology (AMP) have jointly proposed comprehensive standards and guidelines. Known as ACMG/AMP guidelines, these guidelines recommend classifying variants into five distinct categories: "Pathogenic (P)," "Likely Pathogenic (LP)," "Benign (B)," "Likely Benign (LB)," and "Uncertain Significance." The classification is based on 28 evidentiary criteria encompassing functional, case-level genotypic, population allelic, computational, and other data. Each criterion has a designated code, with each code assigned a direction (pathogenic or benign) and an evidence strength weight [1]. While the ACMG/AMP guidelines have facilitated the genetic diagnosis of suspected inherited disorders, primarily Mendelian diseases, further specification and stratification of the evidence criteria are needed. This will address issues related to discordant variant classification resulting from subjective rule interpretations and provide critical flexibility in variant evidence interpretation [2–4].

To address these concerns and refine the ACMG/AMP guidelines, the Clinical Genome Resource (ClinGen), funded by the National Institutes of Health (NIH), established the Sequence Variant Interpretation (SVI) working group. The SVI group has proposed further recommendations for the applying specific criteria, such as PVS1 for loss-of-function (LoF) variants and PP3/BP4 for missense variant based on computational tools [5–7]. These efforts aimed to adapt and enhance the ACMG/AMP guidelines to ensure their precise and consistent application in clinical genetic testing.

One specific pathogenic criterion in the ACMG/AMP guidelines is PS4, designated as 'Strong' level evidence. PS4 refers to a significantly increased prevalence of a variant in affected individuals compared to ancestry-matched controls. It is applicable when the odds ratio (OR) or relative risk (RR) of a variation is > 5.0 and the confidence interval (CI) around the OR/RR does not include 1.0 in case–control comparisons. When an ancestry-matched control population is unavailable, the use of general population data, such as from the Genome Aggregation Database (gnomAD), is recommended to provide evidence for pathogenicity. Alternatively, PS4 evidence for extremely rare variants can be determined by counting the number of probands with the same phenotype, as endorsed by ClinGen SVI. ClinGen has established proband count thresholds for different strength levels of PS4 in various genes and diseases [8–14]. For example, the ClinGen Inherited Cardiomyopathy Expert Panel recommended variants identified in ≥ 2, ≥ 6, or ≥ 15 probands with inherited cardiomyopathies can be assigned supporting, moderate, or strong evidence for PS4, respectively. However, quantitative support for the PS4 thresholds from real-world case–control cohorts is lacking. The absence of defined thresholds could result in misleading variant interpretation and have significant implications for genetic diagnosis and clinical decision-making.

In this study, we addressed this gap by evaluating and defining PS4 thresholds using a dataset of 9,050 variants derived from 13,845 patients with hearing loss (HL) and 6,570 ancestry-matched controls, all part of the Chinese Deafness Genetics Consortium (CDGC) cohort. Genetic HL is a classical Mendelian disease causally linked to thousands of pathogenic variants in over 200 genes, making it an excellent model for assessing PS4 criteria [15–18]. We estimated PS4 thresholds for each strength level by aligning lower boundary values of the positive likelihood ratio (LR^+^) and local positive likelihood ratio (lr^+^) with theoretical values. Furthermore, we validated the accuracy and utility of these defined thresholds by quantifying their impact on variant classification within our extensive patient population.

## Methods

### The Chinese deafness genetics consortium

The CDGC project was initiated in 2013 to investigate the genetic basis of HL and related syndromes. Individuals with moderate-profound HL (pure tone audiometry [PTA] > 40 dB) were recruited by the CDGC workgroup from education programs and medical centers designed for children with hearing loss or deafness across mainland China. Recorded family medical history, perinatal condition, medication history, and other clinical symptoms were gathered through interviewing with the recruited probands and their family members by CDGC investigators. The exclusion criteria were: 1) conductive HL (e.g., HL secondary to otitis media, chronic myringitis, perforated eardrum, and tympanosclerosis); 2) presbycusis (age of onset > 40 years); 3) unilateral HL without a family history; and 4) mild HL without a family history. In addition, controls comprised unrelated adults (≥ 18 years old) without reported hearing impairment, were recruited by the CDGC workgroup and the Fudan Huabiao Project [19].

Blood samples were collected from each participant using anticoagulant vacutainers containing EDTA. DNA was extracted using the MagNA PURE 96 system (Roche, Germany), processed in 96-well plates. Each patient was screened using a SNPscan assay (Shanghai Genesky Biotech, Shanghai, China), including 96 single nucleotide variants (SNVs), 19 insertions/deletions, and three copy number variation (CNV) loci in *GJB2*, *SLC26A4*, and *MT-RNR1* (Additional file 1: Table S1). Subsequently, exons ± 25 flanking bases of 157 HL-related genes were captured and sequenced in genetically undiagnosed patients and all controls (Additional file 1: Table S2), using the CDGC-HL panel based on Agilent SureSelect Target Enrichment. DNA variants were called following the Genome Analysis Toolkit software best practices workflow (Additional file 2: Supplementary Methods) [20].

Pathogenicity of each variant was classified according to the 2018 guidelines (ACMG/AMP-HL) outlined by the Hearing Loss Variant Curation Expert Panel (HL-EP), with several modifications: 1) PS4 was not applied; 2) decrease the weight of PM2 to a supporting level as per the SVI’s recommendation; 3) PP3/BP4 were assigned as moderate for missense variants based on the vote of 13 in silico predictors, including CADD, DVPred, Eigen, FATHMM_MKL, GERP +  + , MetaSVM, MutPred, Polyphen-2, REVEL, PROVEAN, SIFT, SiPhy, and VEST3 (predicted as damaging/benign in more than 80% of available in silico predictors scores); and 4) PM3 was stringently clarified to only consider combinations of different pathogenic variants present in multiple probands for weight calculation (details in Additional file 2: Supplementary Methods). All variants were classified into P, LP, variant of uncertain significance (VUS), LB, and B categories, in the context of patient phenotype, onset age, HL severity, other otologic testing records, family history, and medication history. Putative diagnostic variants were Sanger sequenced for validation. Final diagnostic decisions were made after multidisciplinary panel discussions, incorporating all available evidence.

### Subject and variant inclusion

**Fig. 1:**
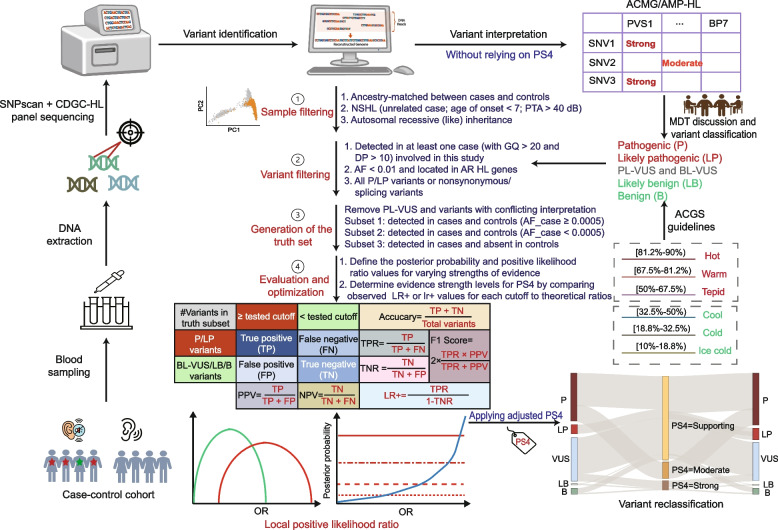
Overview of the Study Design. First, SNPscan assay and HL gene panel sequencing of samples from 22,125 cases and 7,258 controls from the CDGC project were performed. After routine bioinformatic analysis and multidisciplinary panel discussion, variants were classified into five categories: pathogenic (P), likely pathogenic (LP), variants with uncertain significance (VUS), likely benign (LB), and benign (B), based on the ACMG/AMP-HL guidelines, with minor modifications. VUS were further classified into benign-leaning VUS (BL-VUS) and pathogenic-leaning VUS (PL-VUS), according to the ACGS Best Practice Guidelines. Then, samples and variants were filtered to obtain a homogeneous dataset, to minimize biases in defining the PS4 thresholds due to heterogeneity in phenotypes, inheritance patterns, and genetic background. Next, a truth set was generated by removing PL-VUS and variants with conflicting interpretation between CDGC and external databases (DVD, HGMD, and ClinVar). Further, the truth set was divided into three subsets to evaluate and optimize PS4 evidence. For truth subset 1 and 3, positive likelihood ratio (LR^+^) was calculated for each tested cutoff by counting the number of disease-causing and non-pathogenic variant above or below the tested cutoff. In truth subset 2, local positive likelihood ratio (Lr^+^) was estimated by comparing the density ratio of odds ratio (OR) distributions between disease-causing and non-pathogenic variants within a given interval. The lower boundary of LR^+^ /lr^+^ was utilized to determine evidence strength by matching with the thresholds defined for each subset. Finally, the adjusted PS4 criteria were applied in variant reclassification and patient reanalysis. PTA, pure tone audiometry; MDT, multidisciplinary team; GQ, genotype quality; DP, depth; AF_case, AF in cases; TP, true positive (number of P/LP variants above a tested cutoff); FP, false positive (number of BL-VUS/B/LB variants above a tested cutoff); TN, true negative (number of BL-VUS/B/LB variants below a tested cutoff); FN, false negative (number of P/LP variants below a tested cutoff); TPR, true positive rate (sensitivity); TNR, true negative rate (specificity); PPV, positive predictive value; NPV, negative predictive value

A rigorous filtering process was applied to the samples to ensure a homogeneous dataset, minimizing biases in phenotypes, inheritance patterns, and genetic background (Fig. 1 and Additional file 2: Fig. S1). Cases were included if they met the following criteria: 1) clinically diagnosed with non-syndromic HL; 2) absence of conductive HL presbycusis, or unilateral HL; 3) onset of HL before the age of 7; 4) PTA > 40 dB; 5) family history indicating autosomal recessive (AR) inheritance pattern or no reported family history; 6) no molecular diagnosis for autosomal dominant (AD) or syndromic HL genes; 7) no reported or genetically confirmed relationship with any other case or control; and 8) self-reported as Han Chinese.

The inclusion criteria for the control group were as follows: 1) age > 18 years; 2) self-reported normal hearing and normal oral communication; 3) no family history of HL; and 4) self-reported as Han Chinese. Genetic ancestry was inferred using EIGENSOFT/smartpca based on autosomal SNVs with a minor allele frequency (MAF) > 0.001. Outliers identified using smartpca with default parameters were excluded from the analysis [21].

Variants that met all of the following conditions were retained: 1) MAF < 0.01 in controls; 2) genotype quality > 20 and depth > 10; 3) detected in at least one case in this study; 4) located in 66 AR HL genes (Additional file 1: Table S2); and 5) causing nonsynonymous protein coding change (e.g., missense, frameshift, stop gained, in-frame deletion, in-frame insertion, start lost, and stop lost) or potential splicing alterations (within 1–3 bases of the exon or + /–20 bp of the intron boundary), as annotated by the Variant Effect Predictor (VEP) [22]. Additionally, all P/LP variants confirmed by the CDGC project were also included in the analysis.

### Generation of the truth set

A truth set of credibly classified variants, including disease-causing and non-pathogenic variants, was constructed to define the PS4 thresholds. Disease-causing variants comprised P and LP variants identified in cases and classified by the CDGC workgroup. Non-pathogenic variants included B and LB variants, as well as variants classified as "Benign-leaning" VUS (BL-VUS). B and LB variants were classified by the CDGC workgroup or annotated as "Benign" by ClinVar or the Deafness Variation Database (DVD). VUS were categorized into six levels (hot, warm, tepid, cool, cold, and ice cold), according to the Association for Clinical Genomic Science (ACGS) Best Practice Guidelines: 1) hot: 1 strong + 1 supporting or 2 moderate + 1 supporting or 1 moderate + 3 supporting evidence; 2) warm: 1 strong or 2 moderate or 1 moderate + 2 supporting or 4 supporting evidence; 3) tepid: 1 moderate + 1 supporting or 3 supporting evidence; 4) cool: 1 moderate or 2 supporting evidence; 5) cold: 1 supporting evidence; 6) ice cold: no supporting evidence (https://www.acgs.uk.com/quality/best-practice-guidelines/#VariantGuidelines). Variants classified as cool, cold, or ice cold were considered as benign-leaning VUS, unlikely to be disease-causing. Variants classified as hot, warm, or tepid were considered to be pathogenic-leaning VUS. Further, any variants with conflicting interpretation between CDGC, DVD (v9), the Human Gene Mutation Database (HGMD 2022q2), or ClinVar (v20220730) were excluded to ensure the accuracy and appropriate of the truth set.

To address the potential impact of including extremely rare variants that tend to fail to reach statistical significance (p-value < 0.05 or the lower boundary of the 95% CI for the odds ratio [OR_LB] > 1), increasing false negatives, we propose a strategy to improve PS4 threshold assessment. A simulation analysis determined the minimum allele frequency (AF) in cases required for variants where the OR_LB exceeded 1, comparing different case sample sizes (100 to 100,000) to 6,570 controls. Notably, estimating the OR requires a non-zero count of the tested variant in both cases and controls. Therefore, based on the minimum AF in cases achieving significance (min_AF) and the AF in controls (AF_control), the truth set was divided into three subsets: 1) variants present in both groups with AF in cases (AF_case) ≥ min_AF; 2) variants present in both groups with AF_case < min_AF; and 3) variants only present in cases and absent from controls. This approach accounts for the challenges posed by extremely rare variants.

### Define the posterior probability and positive likelihood ratio values for varying strengths of evidence

The evidence strength level was determined using the lower bound of the 95% CI of LR^+^ (LR^+^_LB), following the Bayesian approach proposed by Tavtigian et al. for the 2015 ACMG/AMP guidelines. This ensures that the posterior probability of combined evidence, as outlined in the ACMG/AMP guidelines, is at least 0.9 and less than 0.99 for LP variants, and at least 0.99 for P variants. However, the LR^+^ thresholds suggested by Tavtigian et al. for each strength (2.08 for supportive, 4.33 for moderate, 18.7 for strong, and 350 for very strong) are primarily derived from clinical experience, considering a prior probability of 0.1 [23]. To adapt to the prevalence of disease-causing variants in our dataset, we calculated the posterior probabilities and LR^+^ values for each strength of evidence within the three subsets using the following equations:

1 $${LR}^{+}={C}^{\frac{{N}_{vs}}{1}+\frac{{N}_{s}}{2}+\frac{{N}_{m}}{4}+\frac{{N}_{p}}{8}}$$

2 $$\mathrm{Posterior}\;\mathrm{odds}\;\mathrm{of}\;\mathrm{pathogenicity}=\frac{Odds\;Path\ast prior\;probability}{\left(\left(Odds\;Path-1\right)\ast prior\;probability+1\right)}$$

### PS4 evaluation and optimization

OR (95% CI) and Fisher’s exact p values were calculated for each variant in truth subsets 1 and 2 using R (https://www.R-project.org/). In truth subset 1, variants were deemed enriched in cases if they met: 1) OR > cutoff; and 2) OR_LB > 1. We gradually increased the OR cutoff from 1 to 10. For each cutoff, we computed the following metrics: true positive (TP, the number of P/LP variants above a tested cutoff), false positive (FP, the number of BL-VUS/B/LB variants above a tested cutoff), true negative (TN, the number of BL-VUS/B/LB variants below a tested cutoff), and false negative (FN, the number of P/LP variants below a tested cutoff). Subsequently, we derived LR^+^ , overall accuracy, true positive rate (sensitivity), true negative rate (specificity), positive predictive value (PPV), negative predictive value (NPV), and F1 score from TP, FP, TN, and FN as shown in Fig. 1. Estimates of 95% CI of LR^+^ were generated using bootstrapping in the R package, bootLR [24]. The LR^+^_LB value was utilized to determine evidence strength by matching with the thresholds in Table 1 [23].

**Table 1:** Posterior probability and positive likelihood ratio thresholds for each evidence strength level defined in this study

Truth subset	Variant classification	#Number of variants	Prior probability of pathogenicity	Evidence Strength	Posterior probability of pathogenicity	Positive likelihood ratio
Subset 1		598	0.1338	Very Strong	0.9720	225.0:1
	P/LP	80		Strong	0.6985	15.0:1
	BL-VUS	189		Moderate	0.3743	3.87:1
	B/LB	329		Supporting	0.2331	1.97:1
Subset 2		1997	0.0931	Very Strong	0.9755	387.0:1
	P/LP	186		Strong	0.6689	19.7:1
	BL-VUS	1511		Moderate	0.3130	4.44:1
	B/LB	300		Supporting	0.1778	2.11:1
Subset 3		4951	0.2850	Very Strong	0.9937	397.0:1
	P/LP	1411		Strong	0.8882	19.9:1
	BL-VUS	3370		Moderate	0.6402	4.46:1
	B/LB	170		Supporting	0.4571	2.11:1

In truth subset 2, the lr^+^ value, which is applicable to continuous evidence proposed by Pejaver et al., was estimated to define PS4 thresholds [5]. First, all unique OR values were sorted, then each value was positioned at the center of a sliding window (0.01). The posterior probability was calculated for each OR value within the interval, considering a minimum of 100 disease-causing and non-pathogenic variants. Additionally, the one-sided 95% confidence bound for each estimated lr^+^ was determined through 10,000 bootstrapping iterations, enabling the assessment of evidence strength. The density ratio between OR distributions for disease-causing and non-pathogenic variants within a given interval can be estimated using the following equations:

3 $$Posterior\;probability=\frac{\#P/LP\;variants\;in\;interval}{\#P/LP\;variants\;in\;interval+(Weight\ast\#BL-VUS/B/LB\;variants\;in\;interval)}$$

4 $$Weight=\frac{\left(1-prior\;probability\right)\ast\#P/LP\;variants\;in\;subset\;2}{prior\;probability\ast\left(\#BL-VUS/B/LB\;variants\;in\;subset\;2\right)}$$

In truth subset 3, variant enrichment in cases was assessed based on the case allele count (AC) surpassing a specified cutoff. The cutoff was incrementally raised from 2 to 10. To evaluate the performance, the overall accuracy, sensitivity, specificity, PPV, NPV, F1 score, and LR^+^ values were computed for each cutoff. The LR^+^_LB values were employed to determine the evidence strength levels.

### Independent validation assessing the utility of PS4 thresholds

All variants were reclassified, and genetically undiagnosed patients were reanalyzed using the refined PS4 criteria integrated with existing evidence. For candidate up-graded putative “P/LP” variants and “Hot” VUS, additional phenotypic and pedigree information was collected for identified potential diagnoses, if available. For variants predicted to affect gene splicing, minigene assays were conducted (refer to Additional file 2: Supplementary Methods for details). Reclassified P/LP variants were validated by Sanger sequencing using an ABI 3730xl Genetic Analyzer (Applied Biosystems by Life Technologies, Thermo Fisher Scientific, Inc) and by examining genotype–phenotype co-segregation among family members. A positive genetic diagnosis was assigned when sufficient clinical and genetic evidence was established through multidisciplinary panel discussions.

## Results

### Samples and variants included in this study

**Fig. 2:**
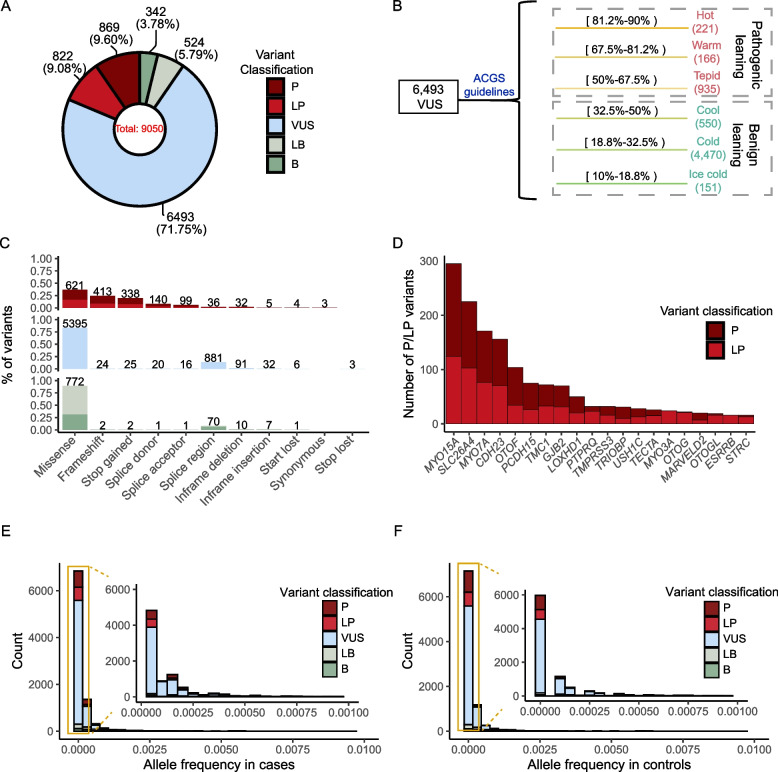
Characteristics of the 9,050 variants included in this study. **A** Summary of classification of the 9,050 variants included in this study. **B** Further classification of VUS to hot, warm, tepid, cool, cold, and ice-cold groups, according to the ACGS Best Practice Guidelines. “Cool/cold/ice cold” variants were defined as benign leaning VUS (BL-VUS), while “hot/warm/tepid” variants were defined as pathogenic leaning VUS (PL-VUS). **C** Distribution of variants by functional impact. **D** Distribution of P/LP variants across the top twenty genes. **E** and **F** Distribution of variant allele frequency in cases and controls

A total of 22,125 HL cases and 7,258 controls from all 31 provincial administrative divisions across mainland China were recruited by the CDGC cohort. The cohort predominantly consisted of cases with early-onset (< 7 yrs, 95.6%), severe/profound (> 70 dB, 94.7%) and non-syndromic (98.8%) HL. Han Chinese was the major ethnic population (17,029, 82.4%). After the filtering based on phenotypes, inheritance patterns, and genetic background, a refined group of 13,845 cases and 6,570 controls fulfilling the criteria were included into this study (Additional file 2: Fig. S1). The included cases had congenital/early-onset hearing impairment (age < 7 years) with moderate-profound HL (PTA > 40 dB), either showing an AR inheritance pattern or lacking family history of HL. After filtering, 9,050 variants across 66 AR HL genes were retained (Additional file 1: Table S3). The CDGC workgroup classified 869 variants as P, 822 as LP, 342 as B, 524 as LB, and 6,493 as VUS (Fig. 2A). Of the VUS, 1,322 were pathogenic-leaning (221 hot, 166 warm, 935 tepid) and 5,171 benign-leaning (550 cool, 4,470 cold, 151 ice cold) (Fig. 2B). Of combined P and LP variants (1,691 in total), 46% (n = 782) had been previously reported in DVD (v9; n = 680), HGMD (2022q2; n = 666), or ClinVar (v20220730; n = 437) [25–27]. Regarding the coding sequence, 83.74% of the P/LP variants were identified, including missense (n = 621, 36.72%), frameshift (n = 413, 24.42%), stop gained (n = 338, 19.99%), in-frame deletion (n = 32, 1.89%), in-frame insertion (n = 5, 0.30%), start lost (n = 4, 0.24%), and synonymous variants (n = 3, 0.18%) (Fig. 2C). Non-coding regions accounted for 16.26% of the P/LP variants, including canonical splice-site alterations (140 splice donor and 99 splice acceptor changes, 14.13%) and splice region changes (n = 36, 2.13%). Top implicated genes with P/LP variants were *MOY15A* (295), *SCL26A4* (225), *MYO7A* (171), *CDH23* (156), *OTOF* (104), *PCDH15* (75), *TMC1* (72), *GJB2* (70), *LOXHD1* (50), *PTPRQ* (32), and *TMPRSS3* (32), collectively encompassing 75.81% of the P/LP variants (Fig. 2D). Among all analyzed variants, 94.77% (n = 8,577) had an AF_control < 0.0007 (PM2_Supporting), and 65.93% (n = 5,967) had an AF_control = 0 (Fig. 2E and F).

### Posterior probability and positive likelihood ratio values for varying strengths of evidence

**Fig. 3:**
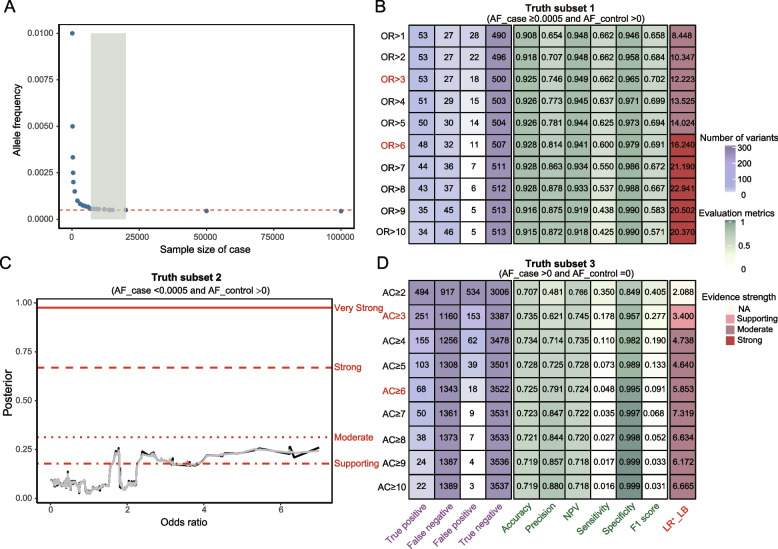
Evaluation and optimization of PS4 in the truth set. **A** Distribution of sample sizes among cases and the corresponding minimum allele frequencies in cases for variants that reached a p-value < 0.05. Based on this result, the truth set was divided into three subsets: subset 1, variants present in both cases and controls with AF in cases ≥ 0.0005; subset 2, variants present in both cases and controls with AF in cases < 0.0005; subset 3, variants present only in cases and absent in controls. Since odds ratio (OR) estimation requires a non-zero count of the tested variant in both the case and control groups, it was only calculated for subset 1 and subset 2 and AC was used for the optimization of cases in subset 3. **B** In truth subset 1, a variant was considered as enriched in cases if the OR was above the given cutoff, the lower bound of the 95% confidence intervals around the estimate of the OR was > 1. **C** Local posterior probability curves of different OR values in truth subset 2. The horizontal lines represent the posterior probability thresholds for supporting, moderate, strong, and very strong evidence. The black curves represent the posterior probability estimated from the truth subset 2. The grey curves represent one-sided 95% confidence intervals calculated from 10,000 bootstrap samples of this dataset. **D** In truth subset 3, variant enrichment in cases was assessed based on the case allele counts above the given cutoff. Numbers of true positive (TP, P/LP variants above a tested cutoff), false negative (FN, P/LP variants below a tested cutoff), true negative (TN, BL-VUS/LB/B variants below a tested cutoff), and false positive (FP, BL-VUS/LB/B variants above a tested cutoff) calls in truth subsets 1 and 3 are shown, along with evaluation metrics, including sensitivity (true positive rate), specificity (true negative rate), accuracy, positive predictive value (PPV, precision), negative predictive value (NPV), F1 score, and the lower boundary of the 95% confidence interval of the positive likelihood ratio (LR^+^_LB)

In total, the truth set comprised 1,677 disease-causing (P/LP) variants and 5,869 non-pathogenic (BL-VUS/LB/B) variants, all of which exhibited consistent interpretations between CDGC and public databases. To reduce false negatives, simulation analysis determined AF thresholds achieving statistical significance. The results, illustrated in Fig. 3A and summarized in Additional file 1: Table S4, revealed a minimum case AF of 0.0005 yielded statistical significance across sample sizes from 7,000 to 20,000. Additionally, estimating OR required a non-zero variant count in both cases and controls. Consequently, the truth set was divided into three subsets: subset 1 included 80 P/LP variants and 518 non-pathogenic (189 BL-VUS and 329 B/LB) variants in both groups with AF_case ≥ 0.0005 and AF_control > 0 (Additional file 1: Table S5); subset 2 included 186 P/LP variants and 1,811 non-pathogenic (1,511 BL-VUS and 300 B/LB) variants with AF_case < 0.0005 and AF_control > 0 (Additional file 1: Table S6); and subset 3 included 1,411 P/LP variants and 3,540 non-pathogenic (3,370 BL-VUS and 170 B/LB) variants only in cases (Additional file 1: Table S7).

For subset 1, we anticipated LR^+^_supporting ≥ 1.97, LR^+^_moderate ≥ 3.87, LR^+^_strong ≥ 15.0, and LR^+^_verystrong ≥ 225.0, corresponding to each pathogenicity evidence strength (Table 1). Additionally, theoretical LR^+^ values for each evidence strength in subset 2 and subset 3 were LR^+^_supporting ≥ 2.11, LR^+^_moderate ≥ 4.44, LR^+^_strong ≥ 19.7 and LR^+^_verystrong ≥ 387.0; and LR^+^_supporting ≥ 2.11, LR^+^_moderate ≥ 4.46, LR^+^_strong ≥ 19.9 and LR^+^_verystrong ≥ 397.0, respectively (Table 1).

### PS4 assessment and refinement

We first evaluated the original ACMG/AMP PS4 guideline in truth subset 1. As recommended, PS4 was assigned as strong evidence based on Fisher’s Exact test OR > 5 and OR_LB > 1 in case–control comparison. As a result, 64 variants were significantly enriched in cases versus controls: 50 P/LP variants, 8 BL-VUS, and 6 B/LB variants (Additional file 1: Table S8). All 50 P/LP variants were previously reported in DVD, HGMD, or ClinVar databases. The accuracy was 0.926, PPV was 0.781, sensitivity was 0.625, specificity was 0.973, and LR^+^ was 23.125 (95% CI, 14.024–43.761), reaching the moderate (LR^+^_LB ≥ 3.87) evidence level.

Next, we refined the PS4 rule by analyzing truth subset 1 with different OR cutoffs. With OR cutoffs from 1 to 5, LR^+^_LB values ranged from 8.448 to 14.024, reaching the moderate level, indicating moderate PS4 evidence (Fig. 3B and Additional file 1: Table S8). The specificity and NPV were generally high (0.944–0.973), but at the expense of lower sensitivity (0.625–0.662) and PPV (0.654–0.781). Notably, all significantly enriched P/LP variants had OR > 3. Of 53 P/LP variants with OR > 3 and OR_LB > 1, 89% (n = 47) had AF_control < 0.0007. With OR cutoffs > 6, the LR^+^_LB (16.240) reached the threshold for strong evidence. Defined PS4 thresholds based on OR and OR_LB, hereafter referred as PS4_OR, are presented in Table 2.

**Table 2:** Summarize the proposed recommendations for the use of PS4 in this study

Rule	Rule Description: The prevalence of the variant in affected individuals is significantly increased compared to the prevalence in controls
PS4	PS4_OR: RR or OR, as obtained from case–control studies, is > 6.0 and the confidence interval around the estimate of RR or OR does not include 1.0
PS4_Moderate	PS4_OR: RR or OR, as obtained from case–control studies, is > 3.0 and the confidence interval around the estimate of RR or OR does not include 1.0; or PS4_AC: allele count ≥ 6 in probands with variant absent in controls
PS4_Supporting	PS4_OR: OR or RR, as obtained from case–control studies, is > 2.27 with variants had an AF < 0.0005 in cases; or PS4_AC: allele count ≥ 3 in probands with variant absent in controls

To determine thresholds for different evidence levels in truth subset 2, we calculated local posterior probabilities for each unique OR score. Scores that satisfied the posterior probability thresholds in Table 1 were considered to provide the corresponding evidence strengths. As shown in Fig. 3C, we identified a threshold for supporting PS4 evidence with OR > 2.27, which had a local posterior probability of 0.180 (95% CI of 0.179).

To define PS4 thresholds for truth subset 3, we utilized case AC to identify variants clustered in cases and absent from controls. AC cutoffs of 2 to 10 were established based on the case AC distribution (Additional file 2: Fig. S2). With AC = 3, the sensitivity was 0.178 and specificity was 0.957, resulting in an LR^+^_LB of 3.400, indicating supporting PS4 evidence could be assigned (LR^+^_LB ≥ 2.11) (Fig. 3D and Additional file 1: Table S8). With AC cutoffs of 4–10, LR^+^_LB ranged from 4.640 to 7.319, surpassing moderate strength. The specificity for these seven AC cutoffs were generally high (0.982–0.999) and NPV were 0.718–0.735. Given achieved a specificity > 99% at AC = 6 (99.5%), and in accordance with the PS4 thresholds for AD HL as proposed by HL-EP, which recommend the assignment of PS4_Moderate to a variant (AF ≤ 0.002%) identified in ≥ 6 probands, we hereby propose the adoption of an AC threshold ≥ 6 to represent moderate evidence strength. The defined PS4 thresholds based on case AC for variants absent in controls, hereafter referred to as the PS4_AC, are presented in Table 2.

The ACMG/AMP guidelines recommend using ancestry-matched subjects from population databases as controls for PS4 when a control population is unavailable. We evaluated the performance using gnomAD (v2.1) population data as controls. Data on AC and total allele number (AN) for each variant in the gnomAD East Asian population (gnomAD_EAS, including 1,909 Koreans, 76 Japanese, and 7,212 other East Asian), considered to have the closest ancestry match to the CDGC patients, were retrieved [28]. Variant AF in gnomAD_EAS and CDGC controls were compared. In total, 1,980 variants were observed in both CDGC controls and gnomAD_EAS, including 132 P/LP variants, 1,267 VUS and 581 B/LB variants. The Pearson correlation coefficient of variant AF between the two populations was 0.517, indicating considerable divergence for rare variants (Additional file 2: Fig. S3). Moreover, OR value correlation was moderate (r = 0.726) between CDGC controls and gnomAD_EAS, underscoring caution when utilizing general population data as controls.

### Utility of the proposed PS4 thresholds in genetic testing

Subsequently, we applied the refined PS4 criteria to all 9,050 variants included in this study, of which 796 variants were tagged with the adjusted PS4: 206 (24% of 869) P variants, 148 (18% of 822) LP variants, 372 (6% of 6,493) VUS, 36 (7% of 524) LB variants, and 34 (10% of 342) B variants (Additional file 1: Table S9). Among 354 summed PS4-tagged P and LP variants, 75% (n = 264) were reported in DVD, HGMD, or ClinVar. Within the 796 PS4-tagged variants, 41.96% (n = 334) were assigned through PS4_OR, and 58.04% (n = 462) were assigned through PS4_AC. Assigned PS4 evidence levels were 61 (7.66%) strong, 108 (13.57%) moderate, and 627 (78.77%) supporting. Notably, 13 variants were upgraded from LP to P. To verify the utility of PS4 in genetic testing, independent evidence including revisiting potential diagnosed cases and experimental analysis were conducted. Significantly, two out of the 372 VUS were validated and reclassified as P/LP variants (*TMPRSS3*: c.205+5G>C and *OTOA*: p.Leu12Arg), which were identified in a total of eight cases (Additional file 1: Table S10).

**Fig. 4:**
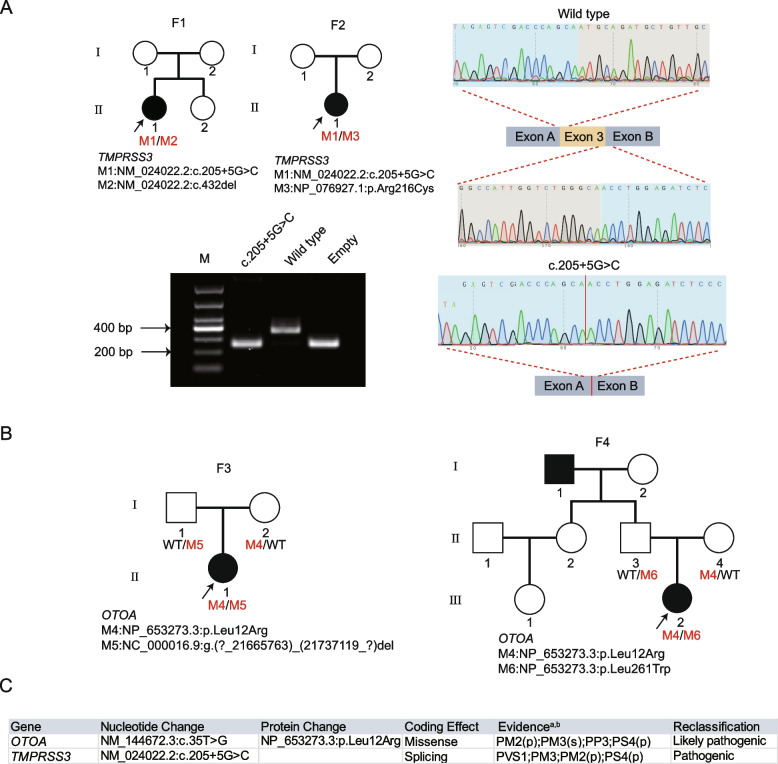
Effect of adding adjusted PS4 on variant reclassification and reanalysis. **A** Pedigrees and molecular features of two probands diagnosed after reanalysis with the adjusted PS4 carrying the *TMPRSS3*: c.205+5G>C variant. Mini-gene splicing assay to investigate the influence of c.205+5G>C on *TMPRSS3* splicing. Agarose gel electrophoresis of RT-PCR products (Lane 1: Marker; Lane 2: 263 bp; Lane 3: 374 bp (263 bp + 111 bp); Lane 4: empty vector (263 bp)) and sequencing results of the mini-gene product, confirming that the *TMPRSS3* mutant mini-gene (c. 205+5G>C) caused a splicing abnormality, resulting in exon 3 skipping. **B** Pedigrees and molecular features of two probands diagnosed after reanalysis with the adjusted PS4 carrying the *OTOA*: p.Leu12Arg variant. **C** Overview of variants upgraded from VUS to P/LP. s: strong; m: moderate; p: supporting; a: PS4 based on proposed optimized thresholds determined in this study; b: evidence strength level was assigned according to the ACMG/AMP-HL guideline recommendations, with minor modification; F1: Family 1; F2: Family 2; F3: Family 3; F4: Family 4

The heterozygous *TMPRSS3*: c.205+5G>C variant was identified in four cases. In two of these cases (Case 1 and Case 2), the c.205+5G>C variant co-occurred with two reported pathogenic alleles (*TMPRSS3*: p.Gln144HisfsTer8 and *TMPRSS3*: p.Arg216Cys), representing moderate level of PM3 [29, 30]. In another two cases (Case 7 and Case 8), c.205+5G>C co-existed with heterozygous VUS (p.Cys204Gly and p.Gly344Arg). This variant was not annotated in either the dbSNP or gnomAD database or our in-house control samples (PM2_Supporting). Several in silico predictive tools, including SpliceAI (0.9873) and MaxEntScan (10.13), indicated the potential splicing impact of this variant at the DNA level (PP3) [31, 32]. Together, the initial evidence strength for *TMPRSS3*: c.205+5G>C was assigned as PM3, PM2_Supporting, and PP3, categorizing it as a “Warm” VUS. Given that the c.205+5G>C variant was heterozygous in four cases (AC = 4) and entirely absent in the control group, PS4_Supporting was assigned, upgrading it to a “Hot” VUS. To assess the splicing effect of c.205+5G>C, wild-type (WT) control and mutant (c.205+5G>C of *TMPRSS3*) mini-genes were constructed. The minigene assays demonstrated that the mutant (c.205+5G>C) caused abnormal splicing and exon 3 skipping (Fig. 4A). In summary, the c.205+5G>C variant was upgraded to pathogenic with the following evidence: PVS1; PM2_Supporting; PM3; and PS4_Supporting. Case 1 and Case 2 received a genetic diagnosis after reanalysis, with further details described in Additional file 2: Supplementary Results.

Additionally, the NM_144672.3: c.35T>G (*OTOA*: p.Leu12Arg) variant was initially identified with an AC_case of 5, found in four patients. It exhibited homozygosity in Case 3 (Family 3, non-consanguineous parents), co-occurrence with a heterozygosity LP variant (*OTOA*: p.Leu261Trp) in Case 4 (Family 4), co-occurrence with a VUS variant in Case 5, and carrier status in the case of Case 6, indicating moderate evidence of PM3. In addition, the c.35T>G variant had supporting evidence of PM2 (AF < 0.0007) and was predicted as damaging by eight tools out of 12 tools (PP3), leading to its classification as a "Warm" VUS. Because of its complete absence in the control population and an AC_case > 3, we introduced PS4 as supporting evidence. Collectively, these criteria warranted the upgrade of this variant to a "Hot" VUS, based on the following supporting evidence: PM2_Supporting; PM3; PP3; PS4_Supporting. We further revisited Family 3 and Family 4 and successfully obtained blood samples from the parents. Subsequent Sanger sequencing revealed compound heterozygosity for *OTOA*: p.Leu12Arg and *OTOA*: p.Leu261Trp in Case 4 (Additional file 2: Fig. S4). In the case of Family 3, Sanger sequencing showed that the father exhibited the wild-type allele, while the mother was found to be heterozygous for the c.35T>G variant (as illustrated in Additional file 2: Fig. S4). Paternity tests provided additional confirmation of their biological relationship. Furthermore, a CNV-seq assay was performed to investigate whether a CNV spanned the genomic region encompassing the c.35T>G variant, potentially explaining the panel sequencing results suggestive of homozygosity. The result revealed that the father carried a heterozygous CNV (NC_000016.9: g.(?_21665763)_(21737119_?)del), while the proband exhibited compound heterozygosity with NM_144672.3:c.35T>G and the NC_000016.9: g.(?_21665763)_(21737119_?)del CNV deletion (Additional file 1: Table S11). Considering this collective evidence, we revised the AC_case of the c.35T>G variant to 4 and upgraded the PM3 evidence to a strong level. Consequently, the c.35T>G variant was reclassified as LP with the following supporting evidence: PM2_Supporting; PM3_Strong; PP3; PS4_Supporting. Genetic diagnoses were established for Case 3 and Case 4 (Fig. 4B and C; further details are provided in the Additional file 2: Supplementary Results).

## Discussion

Since introduction, the ACMG/AMP guidelines have undergone continuous review and refinement for different rules, genes, and diseases, driving optimization and enhancing variant interpretation standards in genetic testing. Here, we estimated PS4 thresholds utilizing ancestry-matched data from 13,845 HL patients and 6,570 controls from the CDGC project, which is, to our knowledge, the largest single-morbidity Mendelian disease cohort available to date. Our results defined supporting, moderate, and strong PS4 evidence thresholds, establishing a foundation for applying PS4 in the genetic testing of HL (summarized in Table 2). Implementing the adjusted PS4 criteria upgraded 13 LP variants to pathogenic, reclassified two VUSs as P/LP, and enabled diagnosis of four additional patients. These results demonstrate the utility of defined PS4 thresholds for improving diagnostic confidence in HL.

In this study, we determined supporting, moderate and strong evidence levels for PS4 by comparing observed LR^+^ or lr^+^ values to theoretical ratios. The ACMG/AMP guidelines recommend assigning strong PS4 to variants with OR (or RR) > 5 and 95% CI of OR (or RR) > 1.0 in case–control comparisons. Our data indicate the recommended OR > 5 threshold is fairly precise, while OR > 6 is required to achieve strong evidence strength based on credibly classified variants. Moreover, we demonstrated that OR > 3 corresponds to moderate evidence. Of P/LP variants identified in our patients, 50 were labeled with strong PS4 evidence according to the guidelines, but all were established HL-causing variants reported in databases and commonly detected in patients. This suggests that applying a stricter threshold has limited utility for interpreting most pathogenic mutations, which are typically rare. To expand the application of PS4 to rare variants, we calculated a local positive likelihood ratio, which revealed that variants with AF_case < 0.0005 could be assigned a supporting PS4 when the OR > 2.27. For variants absent in controls, we propose PS4_AC, which considers variant allele counts across available unrelated cases, to identify variants that cluster in cases, and demonstrated its effectiveness as supporting and moderate evidence. By refining PS4 evidence, we tagged approximately seven times more P/LP variants (354/50), maximizing the power of this evidence in practice, whilst the trade-off was reduced specificity (0.940).

While general population data is valuable for prioritizing pathogenic variants and has been widely utilized for assigning PS4 and PM2 [8–14], caution should be exercised when relying solely on general population data from databases as controls. First, database populations are often categorized continentally or nationally, overlooking fine-grained subpopulation stratification. For instance, East Asians in gnomAD are grouped as "Korean," "Japanese," and "other East Asians". However, considerable genomic difference among Han Chinese, Japanese, and Korean populations were reported, suggesting they should not be treated as a single group in certain applications [33]. Indeed, we observed substantial AF and OR divergence between gnomAD_EAS and CDGC controls despite comparable sample sizes. Similarly, Park et al. reported that OR values for *BRCA1* p.Ser1577Pro, *BRCA2* p.Thr582Pro, and *BRCA2* p.Asp1618Glu, were falsely inflated using ExAC East Asian as controls versus 1,314 in-house Korean controls [34]. Therefore, to improve variant interpretation precision and minimize potential confounding factors from general population data, establishing well-characterized, ancestry-matched control cohorts with adequate number of controls is crucial, especially for underrepresented groups like African Americans and South Asians, which harbor a significant number of unique variants.

Our analysis revealed high correlation between PS4_AC and PM3 (Pearson’s Chi-squared p-value < 2.2e-16), indicating concurrent application of PS4_AC and PM3 should be approached cautiously (Additional file 1: Table S12). Dependency and correlation between evidence rules is not uncommon in ACMG/AMP, as in BS3/BP4, PVS1/PP3, and others [6, 7, 35–37]. The application of ACMG/AMP guidelines, including new algorithms like the Bayesian framework [23], the points-based system [38], and the ABC system [39], assumes independence among the lines of evidence. However, this assumption risks overlap or double-counting, potentially over-estimating combined evidence for or against pathogenicity. Accordingly, challenges persist in effectively combining correlated evidence for accurate variant interpretation, emphasizing the need to further improve the current evidence scoring system in future studies.

The specific PS4 thresholds proposed in this study are recommended for diagnosing Han Chinese patients with AR genetic HL and can be generalizable to all AR HL genes, as they enabled additional patient identification across different HL genes. Additionally, the PS4 thresholds could potentially be adopted for other genetic diseases/genes/populations that exhibit similar attributes to AR HL regarding penetrance and phenocopies. Importantly, we recommend that the PS4 criteria is only of utility for providing evidence toward pathogenicity and not toward benignity, as the FP rate is generally low for all proposed PS4 thresholds whist the FN rates are generally high. This recommendation is consistent with the conclusions drawn in the study by Lucy Loong and colleagues, which emphasized the role of the PM5 metric in providing evidence for pathogenicity rather than benignity [40].

It is important to acknowledge a limitation that the CDGC cohort primarily comprises patients affected by early-onset, severe/profound, and non-syndromic hearing impairment, which is predominantly related to AR genetic HL. Consequently, the dataset is underpowered for statistical analysis of dominant inheritance patterns. Further real-world assessments of AD HL and other diseases/genes are warranted to improve PS4 stratification using the methods outlined in this study.

## Conclusions

In summary, this study highlights the importance of optimizing thresholds for appropriate PS4 criteria application in variant classification. Through quantitative assessments, we established a foundation for utilizing PS4 evidence by categorizing it as supporting, moderate, or strong. Reanalysis using the refined PS4 criteria successfully diagnosed several additional patients affected by different HL genes, demonstrating the value of optimizing this rule. The recommended adjusted PS4 has potential to improve genetic diagnosis and guide the development of specialized recommendations for other rules, advancing variant interpretation in genetic testing.

### Supplementary Information

**Additional file 1: Table S1.** List of 115 SNVs and three CNVs in SNPscan assay. **Table S2.** 157 hearing loss related genes included in the CDGC-HL panel. **Table S3.** 9,050 variants included in this study. **Table S4.** Allele frequency thresholds for variants could reach *p*-value<0.05 with different case sample size. **Table S5.** Variants in truth subset 1. **Table S6.** Variants in truth subset 2. **Table S7.** Variants in truth subset 3. **Table S8.** Evaluation metrics and positive likelihood ratio with different cutoffs in Truth Subset 1 and Truth Subset 3. **Table S9.** Summary of variants meeting the proposed PS4 thresholds. **Table S10.** The clinical and genetic findings of the 8 patients with upgraded P/LP variants. **Table S11.** CNVplex results of F3:II-1. **Table S12.** Correlation between PS4 and PM3 in the truth subset 3.

**Additional file 2: Supplementary Methods. Supplementary Results. Fig. S1.** Flow chart of subjects and variant inclusion. **Fig. S2.** Distribution of allele count in cases for variants in truth subset 3. **Fig. S3.** Pearson correlation of allele frequency (AF) and odds ratio (OR) between CDGC controls and the gnomAD East Asian population. **Fig. S4.** Results of Sanger sequencing.

## Data Availability

The sequencing and clinical data included in this study are not publicly available due to privacy and legal issues. Controlled data access can be applied to the corresponding author (yuanhj301@163.com) upon reasonable request with IRB approval. Please allow up to three months from request to data sharing for regulatory compliance. The code used to calculate the positive likelihood ratio, the local positive likelihood ratio, and to generate the figures in this paper is available on GitHub [41].

## Abbreviations

AC: Allele count
ACMG/AMP: American College of Medical Genetics and Genomics/ Association for Molecular Pathology
AF: Allele frequency
AF_case: AF in cases
AF_control: AF in CDGC controls
AR: Autosomal recessive
B: Benign
BL-VUS: Benign-leaning VUS
CDGC: The Chinese Deafness Genetics Consortium
CI: Confidence interval
FN: False negative
FP: False positive
gnomAD: The Genome Aggregation Database
HL: Hearing loss
LB: Likely Benign
LP: Likely Pathogenic
LR^+^: Positive likelihood ratio
lr^+^: Local positive likelihood ratio
LR^+^ _LB: The lower bound of the 95% CI of LR^+^
NPV: Negative predictive value
OR: Odds ratio
OR_LB: The lower boundary of the 95% CI for the OR
P: Pathogenic
PPV: Positive predictive value
PTA: Pure tone audiometry
TN: True negative
TP: True positive
VUS: Variants with Uncertain Significance
