# Supplementary material for: Quantitative thresholds for variant enrichment in 13,845 cases: improving pathogenicity classification in genetic hearing loss
Source: Genome Med. 2023 Dec 18;15:116. doi: 10.1186/s13073-023-01271-7 (PMC10726519; doi:10.1186/s13073-023-01271-7)
Supplement: Supplementary file 2 — Additional file 2: Supplementary Methods. Supplementary Results. Fig. S1. Flow chart of subjects and variant inclusion. Fig. S2. Distribution of allele count in cases for variants in truth subset 3. Fig. S3. Pearson correlation of allele frequency (AF) and odds ratio (OR) between CDGC controls and the gnomAD East Asian population. Fig. S4. Results of Sanger sequencing. [file 13073_2023_1271_MOESM2_ESM.docx]

**Supplementary Methods**

**Variant identification**

Raw sequencing reads were filtered to obtain clean reads by using Trimmomatic, and fastp was used to evaluate the quality of sequencing data[1, 2]. The cleaned DNA sequencing reads were mapped to the human reference genome hg19 (GRCh37) using the BWA-MEM algorithm[3]. Ambiguously mapped reads (MAPQ <10) and duplicated reads were removed using Samtools[4]. Genomic variants were called following the Genome Analysis Toolkit software best practices[5].

**Variant annotation**

Then, variants were annotated using Variant Effect Predictor (VEP) with supplemental databases, including gnomAD, HGMD, ClinVar, DVD, and dbNSFP[6-11]. The effects of single-nucleotide variants were predicted by 13 in silico predictors, including CADD, DVPred, Eigen, FATHMM_MKL, GERP++, MetaSVM, MutPred, Polyphen-2, REVEL, PROVEAN, SIFT, SiPhy, and VEST3 [12-24].

**Stratification of PM3**

Very strong PM3 was assigned when the variant in consideration was detected in trans with >4 different pathogenic variants; strong was assigned when detected in trans with 2 or 3 different pathogenic variants; moderate was assigned when detected in trans with one pathogenic variant or a homogeneous variant found in two unrelated cases; and a homogeneous variant found in one case was assigned for supporting PM3.

**Pathogenicity curation**

For the P/LP variants reported in DVD, ClinVar, and HGMD, where the original literature was provided, we reviewed the original reports and collected information on the following aspects: 1) clinical information; 2) genotype information; 3) genetic evidence, e.g., familial cosegregation data; and 4) variant classification, including whether the variant classification was made according to the ACMG/AMP-HL guidelines.

Then, we manually curated the pathogenicity of variants reported in DVD, ClinVar and HGMD: 1) pathogenicity annotations conflicted among DVD, ClinVar, HGMD, the original reports and CDGC; 2) the original literature with this variant was not provided; 3) variant-correlated phenotype was irrelevant to HL; 4) MAF was above 0.005 in CDGC control or general populations, except for a few well-known variants that have a relatively high frequency in controls; 5) the original reports showed insufficient pathogenicity evidence, such as no genotype data, lack of clinical data for syndromic HL, or no familial cosegregation data; and 6) variant reclassification according to the ACMG/AMP-HL guidelines is non-pathogenic.

**Minigene construction and analysis**

Wild-type (WT) control and mutant (c.205+5G>C of *TMPRSS3*) mini genes were constructed using the pSPL3 plasmid (Invitrogen Corporation, Carlsbad, CA). PCR was performed in a 50 μL reaction mixture containing 25 μL 2× PrimeSTAR Max Premix (TaKaRa), 30 ng DNA (from F1-II-1), and 0.2 μM forward (TCTGAGTCACCTGGACAACC) and reverse (ATCTCAGTGGTATTTGTGAGC) primers. The empty vector and intron 3 mutation PCR products were double digested using XhoI and NdeI endonucleases at 37°C for 1 h. The insert was then ligated into pSPL3 at 16°C for 4 h using T4 DNA ligase (TaKaRa). Escherichia coli DH5α competent cells were transformed with both the WT control and mutant mini gene constructs. The transformant constructs were then isolated using an Endo-free Plasmid Kit (OMEGA). The WT and mutant vectors were transfected into HEK-293 cells. After 24 h, RNA was extracted, and RT‒PCR (Primer script RT reagent, Takara) was performed. The PCR products for the WT and mutant vectors were analysed on a 2% agarose gel and sequenced.

**Supplementary Results**

**Case 1:** The proband of family 1 (F1) had congenital profound bilateral sensorineural HL, while the parents and younger brother had unimpaired hearing. Genetic analysis revealed two heterozygous variants in *TMPRSS3*: one known pathogenic variant (c.432del) and the c.205+5G>C variant[25]. The c.205+5G>C variant was not annotated in either the dbSNP or gnomAD database or our in-house control samples (PM2_Supporting). The AC for this novel variant was 4, which reached supporting level evidence of PS4. These findings indicate that the c.432del and c.205+5G>C variants cause the phenotype of the Case 1.

**Case 2:** The proband of family 2 (F2) had congenital profound bilateral sensorineural HL, while the parents had normal hearing. The c.205+5G>C variant and c.646C>T (p.Arg216Cys) heterozygous missense mutations of the *TMPRSS3* gene were identified in the Case 2. The c.646C>T variant was a pathogenic variant reported by Miriam Elbracht et al [26]. Sanger sequencing was performed and verified the two variants in the heterozygous state (Fig. 4A and Fig. S4). Therefore, the variants c.205+5G>C and c.646C>T were shown to cause deafness in Case 2.

**Case 3:** Case 3 had profound bilateral sensorineural HL, while her parents had normal hearing. ABR responses showed a threshold of 80 dB. CT and MRI showed no abnormalities of the inner ear, temporal bone or vestibulocochlear nerve in the patient. Genetic analysis identified a heterozygous missense variant (c.35T>G; p.Leu12Arg) and a whole gene deletion of one *OTOA* copy (Additional file 1: Table S11). The variant had supporting evidence for PS4 (AC=4), strong evidence for PM3 (observed in trans with P/LP variants in two unrelated cases), supporting evidence of PM2 (AF<0.0007), and was predicted as damaging by eight tools out of 12 tools (PP3), explaining the phenotype.

**Case 4:** Case 4 had moderate bilateral sensorineural HL. The proband's parents had normal hearing, while the proband's grandfather (F4: I-1) had deafness. The audiograms of the patient showed a U-shaped configuration with moderate middle frequency and mild-to-moderate HL in the low and high frequencies. Compound heterozygous missense variants (p.Leu12Arg;p.Leu261Trp) in *OTOA* were identified in Case 4. The c.782T>G variant was classified as LP, supported by PM2_Supporting, PM3_Strong, and PP3 in our cohort. Segregating analysis confirmed compound heterozygosity for c.782T>G and c.35T>G variants in Case 4 (Fig. S4), indicating cosegregation and their disease-causing roles in pathogenesis.

**References**

1. Bolger AM, Lohse M, Usadel B: **Trimmomatic: a flexible trimmer for Illumina sequence data.** *Bioinformatics* 2014, **30:**2114-2120.

2. Trivedi UH, Cezard T, Bridgett S, Montazam A, Nichols J, Blaxter M, Gharbi K: **Quality control of next-generation sequencing data without a reference.** *Front Genet* 2014, **5:**111.

3. Li H, Durbin R: **Fast and accurate short read alignment with Burrows-Wheeler transform.** *Bioinformatics* 2009, **25:**1754-1760.

4. Li H, Handsaker B, Wysoker A, Fennell T, Ruan J, Homer N, Marth G, Abecasis G, Durbin R, Genome Project Data Processing S: **The Sequence Alignment/Map format and SAMtools.** *Bioinformatics* 2009, **25:**2078-2079.

5. McKenna A, Hanna M, Banks E, Sivachenko A, Cibulskis K, Kernytsky A, Garimella K, Altshuler D, Gabriel S, Daly M, DePristo MA: **The Genome Analysis Toolkit: a MapReduce framework for analyzing next-generation DNA sequencing data.** *Genome Res* 2010, **20:**1297-1303.

6. Liu X, Li C, Mou C, Dong Y, Tu Y: **dbNSFP v4: a comprehensive database of transcript-specific functional predictions and annotations for human nonsynonymous and splice-site SNVs.** *Genome Med* 2020, **12:**103.

7. Karczewski KJ, Francioli LC, Tiao G, Cummings BB, Alfoldi J, Wang Q, Collins RL, Laricchia KM, Ganna A, Birnbaum DP, et al: **The mutational constraint spectrum quantified from variation in 141,456 humans.** *Nature* 2020, **581:**434-443.

8. Azaiez H, Booth KT, Ephraim SS, Crone B, Black-Ziegelbein EA, Marini RJ, Shearer AE, Sloan-Heggen CM, Kolbe D, Casavant T, et al: **Genomic Landscape and Mutational Signatures of Deafness-Associated Genes.** *Am J Hum Genet* 2018, **103:**484-497.

9. Landrum MJ, Lee JM, Benson M, Brown GR, Chao C, Chitipiralla S, Gu B, Hart J, Hoffman D, Jang W, et al: **ClinVar: improving access to variant interpretations and supporting evidence.** *Nucleic Acids Res* 2018, **46:**D1062-D1067.

10. McLaren W, Gil L, Hunt SE, Riat HS, Ritchie GR, Thormann A, Flicek P, Cunningham F: **The Ensembl Variant Effect Predictor.** *Genome Biol* 2016, **17:**122.

11. Stenson PD, Ball EV, Mort M, Phillips AD, Shiel JA, Thomas NS, Abeysinghe S, Krawczak M, Cooper DN: **Human Gene Mutation Database (HGMD): 2003 update.** *Hum Mutat* 2003, **21:**577-581.

12. Bu F, Zhong M, Chen Q, Wang Y, Zhao X, Zhang Q, Li X, Booth KT, Azaiez H, Lu Y, et al: **DVPred: a disease-specific prediction tool for variant pathogenicity classification for hearing loss.** *Hum Genet* 2022, **141:**401-411.

13. Pejaver V, Urresti J, Lugo-Martinez J, Pagel KA, Lin GN, Nam HJ, Mort M, Cooper DN, Sebat J, Iakoucheva LM, et al: **Inferring the molecular and phenotypic impact of amino acid variants with MutPred2.** *Nat Commun* 2020, **11:**5918.

14. Rentzsch P, Witten D, Cooper GM, Shendure J, Kircher M: **CADD: predicting the deleteriousness of variants throughout the human genome.** *Nucleic Acids Res* 2019, **47:**D886-D894.

15. Ioannidis NM, Rothstein JH, Pejaver V, Middha S, McDonnell SK, Baheti S, Musolf A, Li Q, Holzinger E, Karyadi D, et al: **REVEL: An Ensemble Method for Predicting the Pathogenicity of Rare Missense Variants.** *Am J Hum Genet* 2016, **99:**877-885.

16. Ionita-Laza I, McCallum K, Xu B, Buxbaum JD: **A spectral approach integrating functional genomic annotations for coding and noncoding variants.** *Nat Genet* 2016, **48:**214-220.

17. Choi Y, Chan AP: **PROVEAN web server: a tool to predict the functional effect of amino acid substitutions and indels.** *Bioinformatics* 2015, **31:**2745-2747.

18. Shihab HA, Rogers MF, Gough J, Mort M, Cooper DN, Day IN, Gaunt TR, Campbell C: **An integrative approach to predicting the functional effects of non-coding and coding sequence variation.** *Bioinformatics* 2015, **31:**1536-1543.

19. Dong C, Wei P, Jian X, Gibbs R, Boerwinkle E, Wang K, Liu X: **Comparison and integration of deleteriousness prediction methods for nonsynonymous SNVs in whole exome sequencing studies.** *Hum Mol Genet* 2015, **24:**2125-2137.

20. Carter H, Douville C, Stenson PD, Cooper DN, Karchin R: **Identifying Mendelian disease genes with the variant effect scoring tool.** *BMC Genomics* 2013, **14 Suppl 3:**S3.

21. Adzhubei I, Jordan DM, Sunyaev SR: **Predicting functional effect of human missense mutations using PolyPhen-2.** *Curr Protoc Hum Genet* 2013, **Chapter 7:**Unit7 20.

22. Davydov EV, Goode DL, Sirota M, Cooper GM, Sidow A, Batzoglou S: **Identifying a high fraction of the human genome to be under selective constraint using GERP++.** *PLoS Comput Biol* 2010, **6:**e1001025.

23. Garber M, Guttman M, Clamp M, Zody MC, Friedman N, Xie X: **Identifying novel constrained elements by exploiting biased substitution patterns.** *Bioinformatics* 2009, **25:**i54-62.

24. Ng PC, Henikoff S: **SIFT: Predicting amino acid changes that affect protein function.** *Nucleic Acids Res* 2003, **31:**3812-3814.

25. Sang S, Ling J, Liu X, Mei L, Cai X, Li T, Li W, Li M, Wen J, Liu X, et al: **Proband Whole-Exome Sequencing Identified Genes Responsible for Autosomal Recessive Non-Syndromic Hearing Loss in 33 Chinese Nuclear Families.** *Front Genet* 2019, **10:**639.

26. Elbracht M, Senderek J, Eggermann T, Thurmer C, Park J, Westhofen M, Zerres K: **Autosomal recessive postlingual hearing loss (DFNB8): compound heterozygosity for two novel TMPRSS3 mutations in German siblings.** *J Med Genet* 2007, **44:**e81.

**Supplementary Figures**


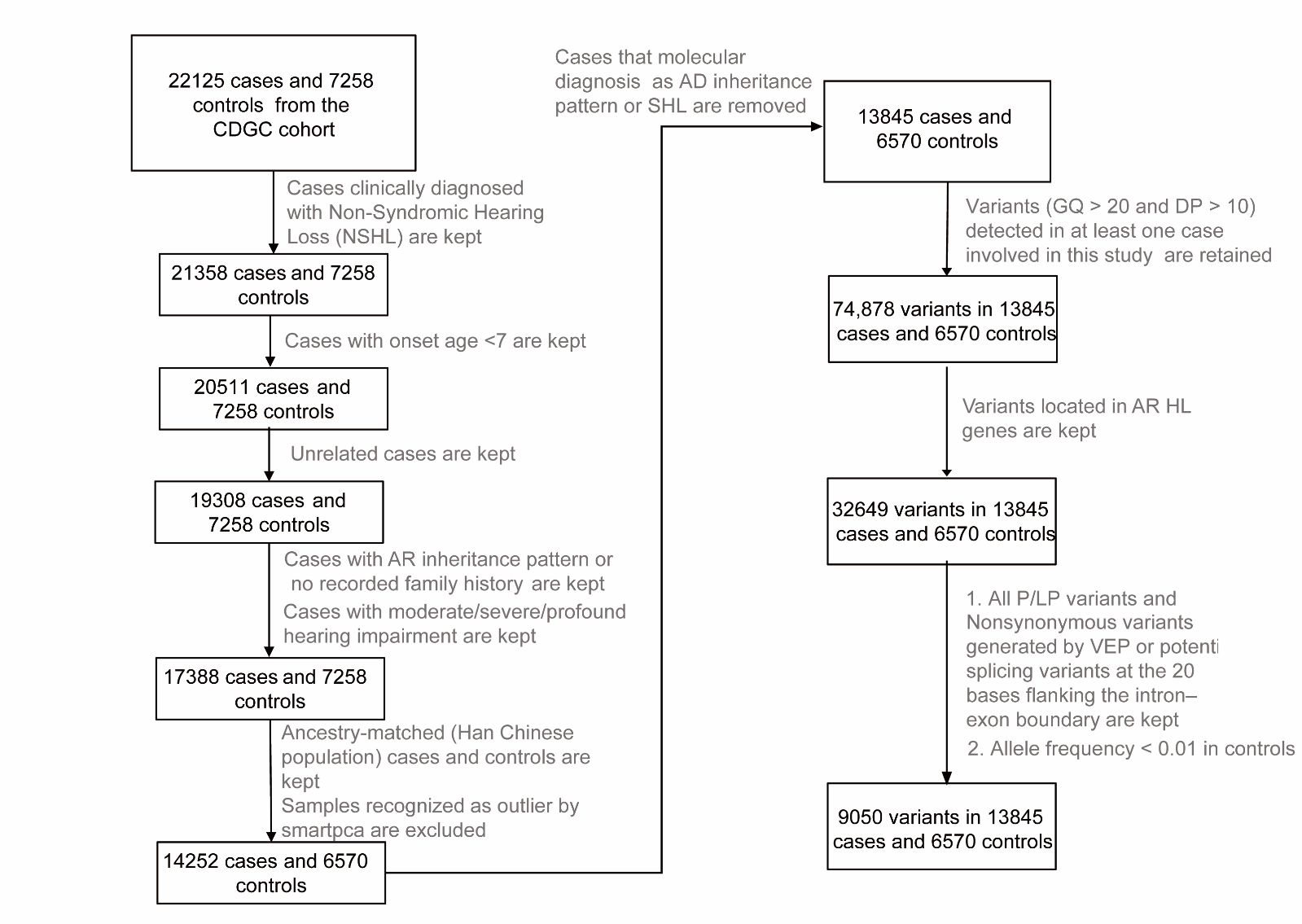


**Fig. S1. Flow chart of subjects and variant inclusion.** AR: autosomal recessive; AD: autosomal dominant; SHL: Syndromic Hearing Loss;GQ: genotype quality; DP: depth.


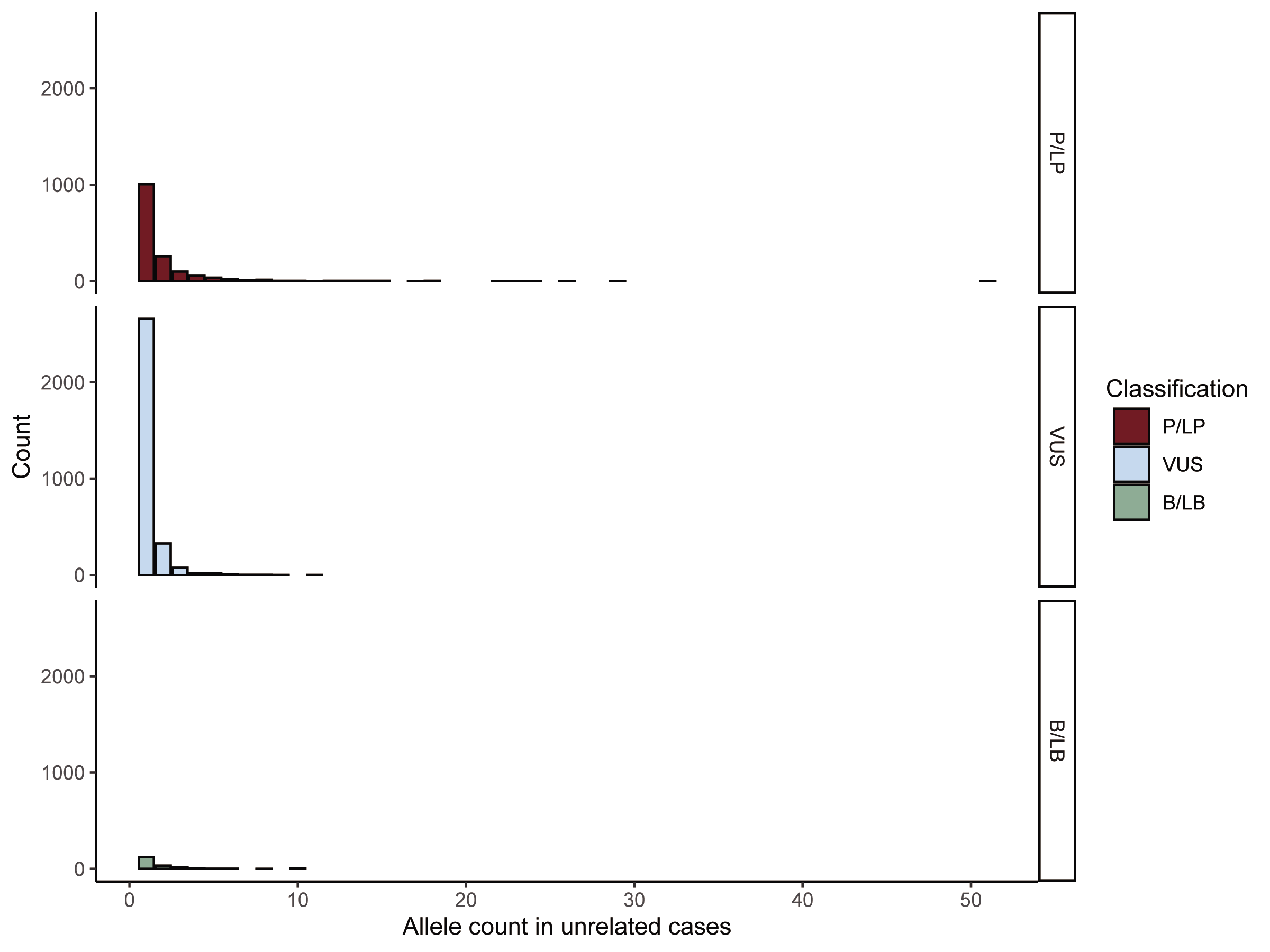


**Fig. S2. Distribution of allele count in cases for variants in truth subset 3.**


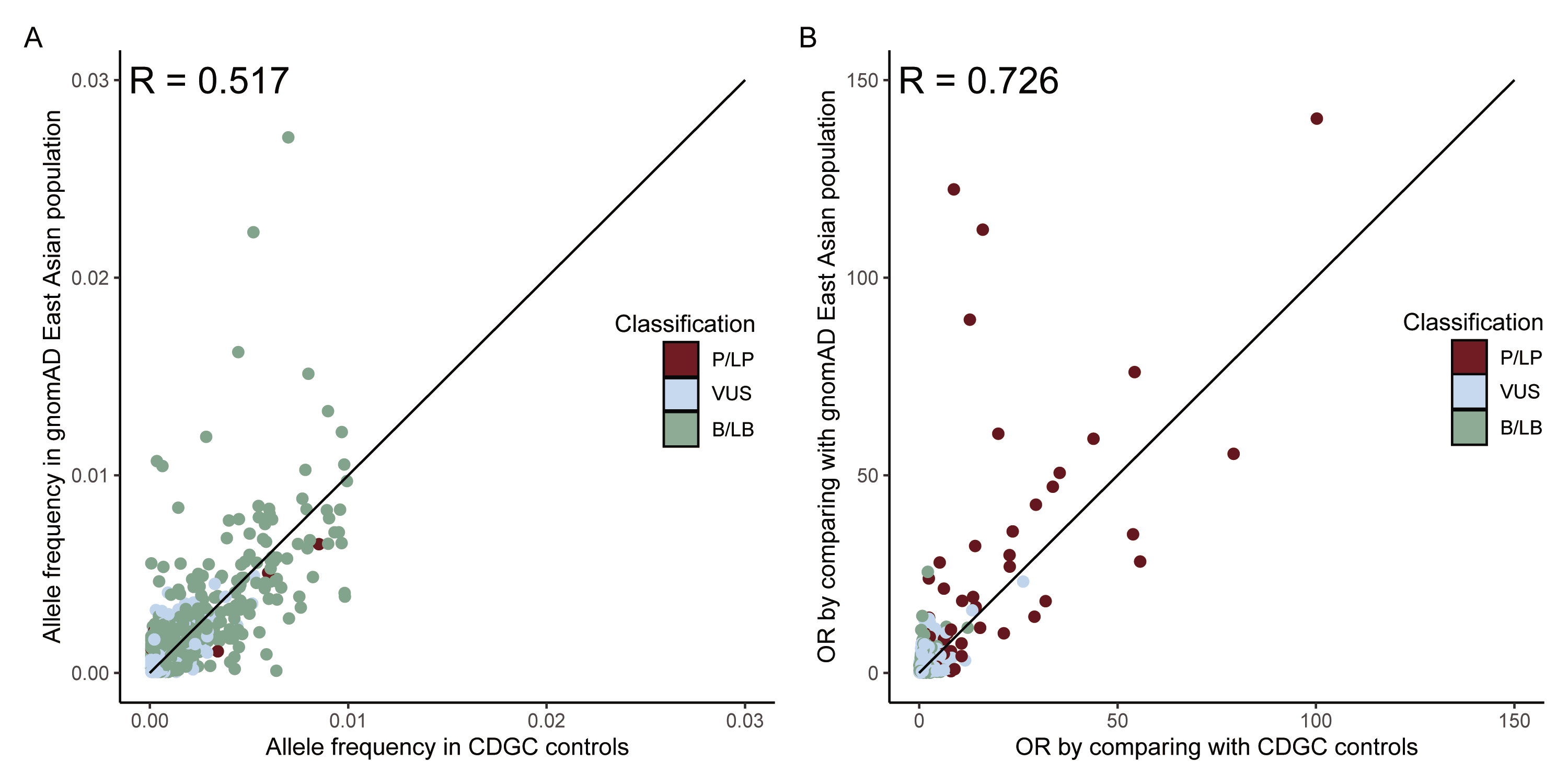


**Fig. S3.** **Pearson correlation of allele frequency (AF) and odds ratio (OR) between CDGC controls and the gnomAD East Asian population.** Red dots indicate P/LP variants. Blue dots indicate VUSs. Green dots indicate B/LB variants.


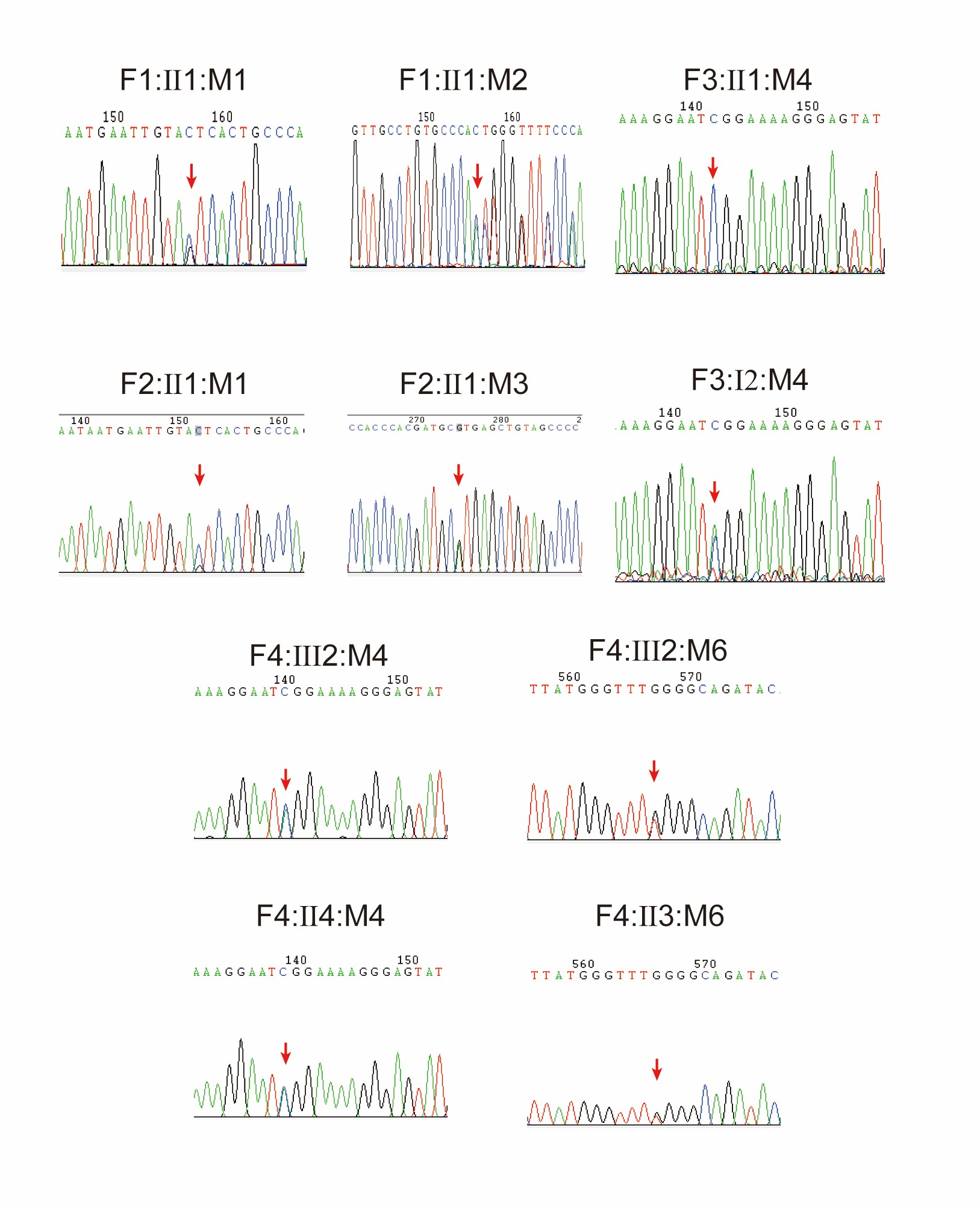


**Fig. S4. Results of Sanger sequencing.** Sanger sequencing traces of *TMPRSS3*: c.205+5G>C and *OTOA*: p.Leu12Arg variants in family 1 (F1), family 2 (F2), family 3 (F3), and family 4 (F4) are presented separately.
